# Knee Kinematics During Curved Walking

**DOI:** 10.7759/cureus.93680

**Published:** 2025-10-01

**Authors:** Masahiko Shimamura, Kenta Horiuchi, Shinya Ogaya

**Affiliations:** 1 Division of Physical Therapy, Graduate School of Health and Social Services, Saitama Prefectural University, Koshigaya, JPN; 2 Department of Physical Therapy, Graduate School of Health and Social Services, Saitama Prefectural University, Koshigaya, JPN

**Keywords:** elderly, gait, kinematic, knee angle, knee rotation

## Abstract

Objective

Walking in a straight line is considered the simplest form of locomotion for healthy individuals. However, in daily life, the ability to walk along curved paths is essential for tasks such as avoiding obstacles and turning corners. Evaluating gait patterns that involve directional changes along curved trajectories offers a more ecologically valid assessment of functional mobility, thereby contributing to the development of rehabilitation strategies that are better aligned with daily living activities. The present study aimed to investigate differences in knee joint rotational angles between straight and curved walking in older adults, using the point cluster technique. Furthermore, the study sought to determine whether distinct knee rotational patterns exist between gentle and sharp curved walking, both categorized under curved walking.

Methods

A total of 40 community-dwelling older adults participated in the study. Each participant performed one trial of straight walking, gentle curve walking (with a curvature radius of 2 meters), and sharp curve walking (with a curvature radius of 1 meter). During each walking condition, reflective markers attached to the body were recorded at a sampling frequency of 100 Hz. From the recorded trajectories of the reflective markers, knee joint angles for flexion-extension, abduction-adduction, and internal-external rotation were calculated.

Results

The maximum flexion angle was 45.3 ± 10.1° during straight walking, 19.1±9.8° during gentle curve walking, and 23.5 ± 11.7° during sharp curve walking, with a significant difference observed by ANOVA (p < 0.01). Additionally, post hoc multiple comparisons showed significant differences between straight walking and gentle curve walking (p < 0.01), straight walking and sharp curve walking (p<0.01), and gentle curve walking and sharp curve walking (p < 0.05).

The maximum external rotation angle was 8.4 ± 7.17° during straight walking, −2.3 ± 7.6° during gentle curve walking, and −5.8 ± 8.2° during sharp curve walking, with a difference observed by ANOVA (p < 0.05). Additionally, post hoc multiple comparisons showed significant differences between straight walking and gentle curve walking (p < 0.05).

Conclusion

During walking along a curved path, individuals adopt a gait strategy that involves internally rotating the knee joint prior to entering the curve. The internal rotation angle of the knee joint during the early stance phase was greater in sharp curve walking than in gentle curve walking, indicating that a smaller curvature radius requires a larger internal rotation angle. These findings suggest that curved walking demands a greater range of knee joint rotation compared to straight walking.

## Introduction

In gait analysis, many studies focus on walking movements under constrained conditions, such as laboratory-based walk-throughs or treadmill walking, which capture only partial aspects of daily life. Straight walking is typically the primary task examined. While walking along a straight path is the simplest locomotor task for healthy individuals, the ability to perform curved walking is essential in daily life for avoiding obstacles and corner turns. In fact, we perform over 800 turns per day on average, amounting to more than 4,500 per week [1] and over 50% of daily steps are reported to involve turning [2]. In elderly persons [3] who exhibit reduced knee rotational range of motion during gait, turning movements often cause difficulty.

Previous research on non-straight walking has examined turning movements such as 180-degree turns [4], and 90-degree turns [5], involving transitions from a straight walking to another trajectory. In contrast in daily life, humans frequently change their walking direction gradually along curved paths while maintaining their gait speed. It has been reported that during walking along a curved path, the activity of the lateral gastrocnemius muscle in the lateral lower limb decreases [6]. Although curved walking involves mechanically induced rotational movements, studies reporting on knee joint rotational movements are limited. Therefore, focus on measuring walking along curved paths has social significance because it enables the evaluation of gait more closely resembling daily life movements, which in turn allows for the proposal of rehabilitation programs better suited to the activities of daily life.

The incidence of meniscal injury increases with age, with the posterior horn medial meniscus being the most frequently affected site [7]. Studies using MRI have reported that during knee flexion, both the medial and lateral menisci shift posteriorly as the degree of flexion increases [8]. Furthermore, research employing finite element methods has shown that the shear stress on the posterior horn of the medial meniscus increases with greater flexion angles [9]. Consequently, movements involving substantial external rotation may elevate the risk of pain originating from the posterior horn medial meniscus. During curved walking, the change in direction requires a shift in body orientation, which is expected to force the outer lower limb to undergo greater external knee rotation during the stance phase. Therefore, it is hypothesized that the greater the curvature of the walking path, the larger the external rotational motion at the knee joint. However, few studies have reported on knee-on-knee rotational kinematics during curved gait.

In particular, older adults tend to exhibit a reduced knee joint range of motion [10], slower gait speed, and shorter step length compared to younger individuals [11]. These pronounced age-related changes in physical function confer substantial clinical relevance, including potential applications in disability prevention and rehabilitation.

Since the rotational movement of the knee occurs within a limited range of motion, its measurement using reflective markers and infrared cameras had technical issues of decreased accuracy due to soft tissue artifacts at the marker placement sites. To reduce the influence of such artifacts, Andriacchi et al. proposed the point cluster technique (PCT), which calculates joint angles using multiple markers attached to the thigh and shank [12]. This technique is considered capable of accurately estimating rotational angles.

Therefore, the aim of this study was to investigate differences in knee joint rotational kinematics between straight and curved walking in older adults, using the PCT for measurement. 

## Materials and methods

Participants

A total of 40 older adults living in the local community were recruited for this study. The measurement data were collected from August 29 to 31, 2023. The participants were gathered at Saitama Prefectural University, and the measurements were conducted within the university's facilities.

The participants had a mean age of 75.0 ± 4.8 years, a mean height of 1.57 ± 0.07 m, and a mean body weight of 56.7 ± 9.6 kg. The inclusion criteria were as follows: (i) aged 65 years or older; (ii) able to live independently; (iii) no history of knee surgery; (iv) no musculoskeletal disorders affecting gait; and (v) no neurological disorders.

Prior to data collection, all participants received a verbal explanation of the study’s objectives and procedures and provided written informed consent. This study was approved by the Ethics Committee of the Affiliated Institution (Approval No. 23102).

Motion measurement

**Figure 1:**
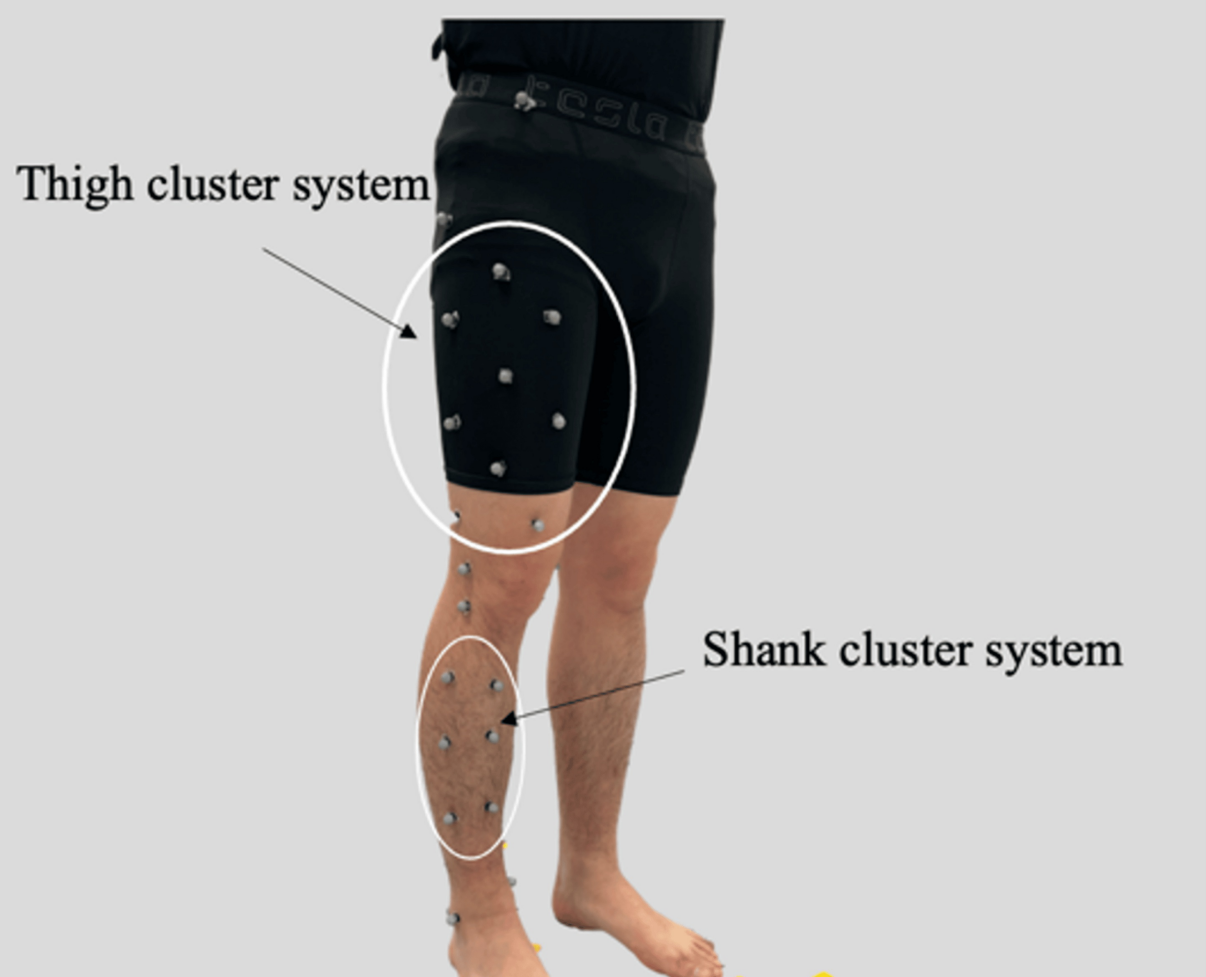
Lower limb marker set. This figure was created by the author.

Kinematic measurements were conducted on the right lower limb during gait using four infrared cameras (OptiTrack, Natural Point Inc., USA) at a sampling frequency of 100 Hz. Reflective markers were attached to the surface of the participants' bodies. The marker set is shown in Figure 1.

To construct anatomical coordinate systems, three bony landmarks of the femur (the greater trochanter, lateral epicondyle, and medial epicondyle) and four bony landmarks of the tibia (the lateral condyle, medial condyle, lateral malleolus, and medial malleolus) were identified and marked with reflective markers. Reflective markers for constructing the cluster system were attached to the skin surface at nine points on the thigh (thigh marker system) and six points on the shank (shank marker system). Additionally, to determine initial contact, markers were placed on the bony landmark of the anterior superior iliac spine and the lateral aspect of the calcaneus. In motion capture-based motion analysis, errors may occur due to marker displacement or skin movement. To minimize these errors, markers were accurately placed on anatomical landmarks, and care was taken for each participant to ensure that marker placement did not introduce any inaccuracies.

**Figure 2:**
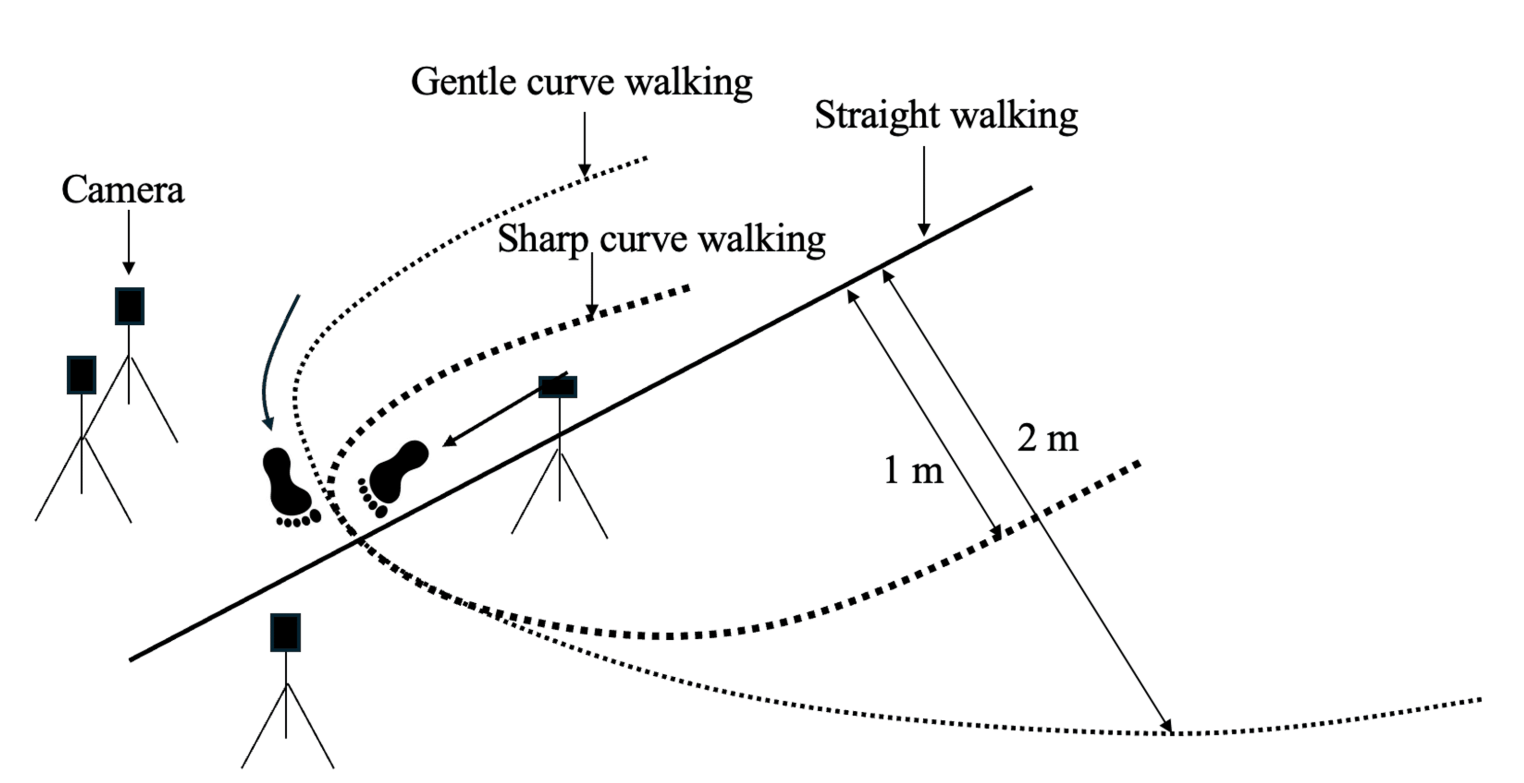
Walking path for gait measurement. This figure has been created by the author. Straight walking (solid line), gentle curve walking (dotted line), and sharp curve walking (bold dotted line). For the straight walking trial, a 2 m approach pathway was provided before the 5 m data collection walkway. Participants were instructed to walk along the curved path in a counterclockwise direction so that the right lower limb being measured was positioned on the outside of the curve. Measurements were conducted on semicircular walking paths with radii of curvature of 1 m and 2 m from the center of curvature, with an approach distance of 1.5 m provided. All trials were performed barefoot by the participants at a self-selected comfortable walking speed. The analysis window focused on the stance phase from initial contact to toe-off at the apex of the radius of curvature.

Initially, a static standing posture was recorded to define the initial position. Subsequently, one trial each of straight walking and gentle curve walking were performed. The walking course used for the measurements is illustrated in Figure 2. For the straight walking trial, a 2 m approach pathway was provided before the 5 m data collection walkway. Participants were instructed to walk along the curved path in a counterclockwise direction so that the right lower limb being measured was positioned on the outside of the curve. Measurements were conducted on semicircular walking paths with radii of curvature of 1 m and 2 m from the center of curvature, with an approach distance of 1.5 m provided. All trials were performed barefoot at the participants and self-selected comfortable walking speed. The analysis window focused on the stance phase from initial contact to toe-off at the apex of the radius of curvature. In daily life, human walk while freely varying their gait speed and stride length. Therefore, evaluation under natural walking conditions is important. For this reason, this study did not measure walking parameters such as gait speed or stride length.

Calculation of the knee internal-external rotational angle

Knee angles were calculated based on the methodology proposed by Andriacchi et al [12]. First, for the cluster systems attached to the thigh and shank segments, the position vector $P(t)_i$ from the center of mass $c(t)$ to each marker $g(t)_i$ was defined as follows:

$P(t)_i = g(t)_i - c(t)$

Here,  $i$ and $t$ represent the marker number and time steps, respectively. The inertia tensor ${I(t)}$ of the marker coordinates was computed from the positions $p$ of each marker $i$ as expressed by the following equation.

\( I(t) = \begin{bmatrix} \sum \left( p_{i,y}^2 + p_{i,z}^2 \right) & \sum p_{i,x} p_{i,y} & \sum p_{i,x} p_{i,z} \\ \sum p_{i,x} p_{i,y} & \sum \left( p_{i,z}^2 + p_{i,x}^2 \right) & \sum p_{i,y} p_{i,z} \\ \sum p_{i,x} p_{i,z} & \sum p_{i,y} p_{i,z} & \sum \left( p_{i,x}^2 + p_{i,y}^2 \right) \end{bmatrix} \)

By calculating the eigenvectors $E_j \quad (j=1,2,3)$ of the moment of inertia tensor $I(t)$, the rotation matrix that transforms each segment into its principal axis system can be determined for both the thigh and the shank coordinate systems.

$R(t) = \begin{bmatrix} E_1(t) & E_2(t) & E_3(t) \end{bmatrix}$

Subsequently, the coordinates in the principal axis system were transformed into the segment coordinate system $P_i(t)$ using the following equation:

$P(t)_i = R(t) \cdot I(t)_i$

$I(t)$ represents the position of the reference point in the principal axis system of inertia. The three orthogonal axes generated by the rotation matrix $R(t)$ represent the principal axes of inertia of the cluster points. Furthermore, to minimize the error between each marker position on the principal axes of inertia and its position on the inertial axes in the static state, skin motion error was reduced by optimizing virtual weights assigned to each marker such that the variation in the eigenvalues of the inertia tensor was minimized.

Once the skin motion artifacts affecting the cluster markers were corrected for each time steps, the anatomical coordinate systems of the femur and tibia during movement were calculated using the reflective markers of the cluster system. Following the joint coordinate system proposed by Grood and Suntay [13], the knee joint angles flexion-extension, abduction-adduction, and internal-external rotation during the stance phase were ultimately calculated. The zero angle was defined as the angle in the upright posture during static standing.

To construct anatomical coordinate systems, three bony landmarks of the femur (the greater trochanter, lateral epicondyle, and medial epicondyle) and four bony landmarks of the tibia (the lateral condyle, medial condyle, lateral malleolus, and medial malleolus) were identified and marked with reflective markers. Reflective markers for constructing the cluster system were attached to the skin surface at nine points on the thigh (thigh marker system) and six points on the shank (shank marker system). Additionally, to determine initial contact, markers were placed on the bony landmark of the anterior superior iliac spine and the lateral aspect of the calcaneus.

Statistical analysis

A one-way analysis of variance (ANOVA) was conducted to compare joint angles across straight walking, gentle curve walking, and sharp curve walking conditions. When significant differences were observed, post-hoc pairwise comparisons between trials were performed using the Bonferroni correction. When significant differences were observed, post-hoc pairwise comparisons between trials were performed using Bonferroni correction. After correction, p < 0.05 was considered significant, and p < 0.01 was considered highly significant. Python (v3.12.7) was used for statistical analysis.

## Results

**Figure 3:**
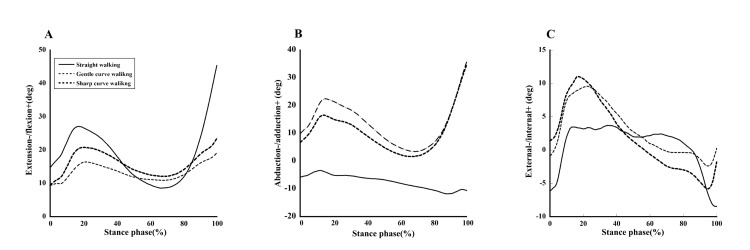
Joint angles during the stance phase in straight, gentle curve, and sharp curve walking. A. Extension and flexion angle, B. Abduction and adduction angle and C. External and internal angle. The joint angles for each walking path are shown as follows: straight walking (solid line), gentle curve walking (dotted line), and sharp curve walking (bold dotted line).

Figure 3 shows the knee flexion-extension, adduction-abduction, and internal-external rotation angles during the stance phase for straight walking, gentle curve walking, and sharp curve walking conditions, and Table 1 shows the maximum angle, minimum angle, and excursion.

**Table 1:** Maximum and minimum joint angles and excursion (mean ± SD) in each walking condition. Stw: Straight walking; Gcw: Gentle curve walking, and Scw: Sharp curve walking; p-values were calculated using one-way ANOVA to assess statistical differences between groups; post hoc comparisons between all test conditions were done with Bonferroni-adjusted p-values. Significance level: 0.05 for all comparisons. ^a^Between condition straight walking (Stw) and gentle curve walking (Gew); ^b^Between condition straight walking (Stw) and sharp curve walking (Swc); ^c^Between condition gentle curve walking (Gcw) and sharp curve walking (Swc).

Angle		Stw	Gcw	Scw	p-values
Flexion+ /Extension- (degrees)	Max	45.3 (± 10.1)^ab^	19.1 (± 9.8)^ac^	23.5 (± 11.7)^bc^	<0.01**
	Min	8.5 (± 7.5)	9.3 (± 6.5)	9.3 (± 6.9)	0.90
	Excursion	36.7 (± 8.2) ^ab^	9.8 (± 8.3)^ ac^	14.0 (± 7.45) ^bc^	<0.01**
Adduction+ /Abduction- (degrees)	Max	-3.3 (± 5.7)^ab^	35.7 (± 9.5)^a^	23.5 (± 11.7)^b^	<0.01**
	Min	-11.7 (± 6.5)^ ab^	3.4 (± 7.3)^a^	1.6 (± 7.6)^b^	<0.01**
	Excursion	8.4 (± 5.5) ^ab^	32.2 (± 4.7)^a^	32.9 (± 9.3)^b^	<0.01**
Internal+ /External- (degrees)	Max	3.8 (± 7.1)^b^	9.5 (± 7.6)	11.0 (± 8.2)^b^	0.01*
	Min	8.4 (± 7.6)	2.3 (± 8.7)	5.8 (± 9.2)	0.13
	Excursion	4.7 (± 5.3)	7.1 (± 5.6)	5.2 (± 7.3)	0.12

The maximum flexion angle was 45.3 ± 10.1° during straight walking, 19.1 ± 9.8° during gentle curve walking, and 23.5 ± 11.7° during sharp curve walking, with a significant difference observed by ANOVA (p < 0.01). Additionally, post-hoc multiple comparisons showed significant differences between straight walking and gentle curve walking (p < 0.01), straight walking and sharp curve walking (p < 0.01), and gentle curve walking and sharp curve walking (p < 0.05). The flexion excursion was 36.7 ± 8.2° during straight walking, 9.8 ± 8.3° during gentle curve walking, and 14.0 ± 7.4° during sharp curve walking, with significant differences observed by ANOVA (p < 0.01). The maximum flexion angle was 45.3 ± 10.1° during straight walking, 19.1 ± 9.8° during gentle curve walking, and 23.5 ± 11.7° during sharp curve walking, with a significant difference observed by ANOVA (p < 0.01).

The maximum adduction-abduction angles were -3.3 ± 5.7° during straight walking, 35.7 ± 9.5° during gentle curve walking, and 3.5 ± 11.7° during sharp curve walking, with significant differences observed by ANOVA (p < 0.01). Additionally, post-hoc multiple comparisons showed significant differences between straight walking and gentle curve walking (p < 0.01), and straight walking and sharp curve walking (p < 0.01). The minimum adduction-abduction angle was −11.7 ± 6.5° during straight walking, 3.4 ± 7.6° during gentle curve walking, and 1.6 ± 7.6° during sharp curve walking, with significant differences observed by ANOVA (p < 0.01). Additionally, post-hoc multiple comparisons showed significant differences between straight walking and gentle curve walking (p < 0.01), and straight walking and sharp curve walking (p < 0.01). The adduction-abduction excursion was 8.4 ± 5.5° during straight walking, 32.2 ± 4.7° during gentle curve walking, and 32.9 ± 9.3° during sharp curve walking, with significant differences observed by ANOVA (p < 0.01). Additionally, post-hoc multiple comparisons showed significant differences between straight walking and gentle curve walking (p < 0.01), and between straight walking and sharp curve walking (p < 0.01).

The maximum external rotation angle was 8.4 ± 7.17° during straight walking, −2.3 ± 7.6° during gentle curve walking, and −5.8 ± 8.2° during sharp curve walking, with a difference observed by ANOVA (p < 0.05). Additionally, post-hoc multiple comparisons showed significant differences between straight walking and gentle curve walking (p < 0.05).

## Discussion

In this study, we compared knee joint internal rotation during walking along straight and curved paths, clarifying the distinct movement patterns exhibited in each condition. During both types of curve walking, the knee angle showed an internally rotated position at the loading response phase and subsequently moved to an externally rotated position from mid-stance. When walking counterclockwise along a curved path, the lateral lower limb contacted the ground with a more internally rotated knee position compared to straight walking. Moreover, this internal rotation was more prominent during the loading response phase, with knee internal rotation reaching approximately 10° and 11° during gentle and sharp curve walking, respectively (Figure 3C).

The results observed during the early stance phase contradicted the hypothesis, which proposed that knee external rotation increases during the stance phase of curve walking. It is presumed that, during walking along a curved path, individuals rotate the knee joint internally at foot contact as part of their gait strategy. Wang et al. reported that during a spin turn, the supporting leg acts as a pivot, and the body rotates while the knee internally rotates. The knee joint moves from an external rotation angle of 15.1° at initial contact toward internal rotation [14]. It is postulated that ground contact, in an externally rotated knee position, facilitates an increased range of motion for knee internal rotation. Although there are differences in task motions between previous studies on turning and the present study on curve walking, it is likely that the knee joint is pre-rotated toward the direction of progression before foot contact, to ensure the alignment for stance. The internal rotational range of motion of the knee in the lower limb appears to be important during curve walking, and age-related restrictions in this rotational range of motion may limit the ability to perform curve walking efficiently.

Comparing the knee internal rotation angles between gentle curve walking and sharp curve walking, an increase of 3.5° was observed in sharp curve walking during the early stance phase. A biomechanical study of the knee has reported that the anterior cruciate ligament, medial collateral ligament, and lateral collateral ligament lose tension during knee flexion, resulting in an increased range of rotational motion [15]. The knee flexion angle during sharp curve walking increased by 5° compared to gentle curve walking. The result suggests that the anatomical structure of the knee joint facilitated the increased internal rotation observed in sharp curve walking.

Regarding the rotational angle from mid-stance to toe-off, straight walking exhibited a gradual external rotation, whereas curve walking showed greater changes in external rotation angle. A previous study, walking along a sinusoidal walking path [16], reported increased knee external rotation during the late stance phase, when walking with the lateral lower limb as the pivot. This finding is consistent with the results of the present study. Knee external rotation may contribute adaptively to adjusting the direction of progression and to aligning the body along the path during curve walking.

A potential limitation of the present study is that opinions differ regarding the validity of the measurement accuracy of the PCT [17-19], which imposes a constraint on the interpretation of the measurement results. Regarding the observed increase in the knee adduction angle during the latter stance phase (after 80% of the gait cycle) in walking along a curved path, it is possible that this was influenced by trunk and lower limb rotation when passing through a corner. The rotation may have caused the reflective markers, comprising the cluster attached to the femur, to be pulled toward the medial and lateral sides of the thigh, resulting in asymmetric movement of the reflective markers. Another limitation of this study is that walking speed was not measured. It has been reported that an increase in walking speed leads to an increase in knee joint external rotation angle [20]. In this study as well, higher walking speeds may have caused an increase in knee joint external rotation.

For walking rehabilitation and disability prevention, it is also necessary to include diverse gait assessments that can evaluate curved walking, which imposes external rotation on the knee. This is particularly useful for assessing the gait patterns of patients with a reduced knee external rotation range of motion or pain caused by external rotation.

## Conclusions

In this study, we investigated knee joint internal rotation during walking along straight and two curved paths, clarifying the movement patterns for curve walking. The results of the curve walking showed that the knee joint exhibited internal rotation at the loading response phase and then rotated externally until mid-stance. Notably, during the loading response phase (around 20% of the gait cycle), the internal rotation angles of the knee joint in gentle curve walking and sharp curve walking showed a clear increase compared to straight walking. On the other hand, no significant differences were observed in the external rotation angles. Ground contact in an externally rotated knee position was possible to expand the range of motion available for knee internal rotation. Although there are differences in task motions between previous studies on turning and the present study on curve walking, it is likely that the knee joint is pre-rotated toward the direction of progression before foot contact to ensure the alignment for stance.

## References

[1] Fuller JR, Adkin AL, Vallis LA (2007). Strategies used by older adults to change travel direction. Gait Posture.

[2] Glaister BC, Bernatz GC, Klute GK, Orendurff MS (2007). Video task analysis of turning during activities of daily living. Gait Posture.

[3] Boyer KA, Andriacchi TP (2016). The nature of age-related differences in knee function during walking: implication for the development of knee osteoarthritis. PLoS One.

[4] Stein RB, Hase K (1999). Stopping and turning during human walking. Prog Brain Res.

[5] Taylor MJ, Strike SC, Dabnichki P (2006). Strategies used for unconstrained direction change during walking. Percept Mot Skills.

[6] Courtine G, Schieppati M (2003). Human walking along a curved path. II. Gait features and EMG patterns. Eur J Neurosci.

[7] Tsujii A, Nakamura N, Horibe S (2017). Age-related changes in the knee meniscus. Knee.

[8] Bylski-Austrow DI, Ciarelli MJ, Kayner DC, Matthews LS, Goldstein SA (1994). Displacements of the menisci under joint load: an in vitro study in human knees. J Biomech.

[9] Yokoe T, Ouchi K, Yamaguchi Y, Enzaki M, Tajima T, Chosa E (2023). Shear stress in the medial meniscus posterior root during daily activities. Knee.

[10] Kefala V, Cyr AJ, Harris MD, Hume DR, Davidson BS, Kim RH, Shelburne KB (2017). Assessment of knee kinematics in older adults using high-speed stereo radiography. Med Sci Sports Exerc.

[11] Olney SJ, Griffin MP, McBride ID (1994). Temporal, kinematic, and kinetic variables related to gait speed in subjects with hemiplegia: a regression approach. Phys Ther.

[12] Andriacchi TP, Alexander EJ, Toney MK, Dyrby C, Sum J (1998). A point cluster method for in vivo motion analysis: applied to a study of knee kinematics. J Biomech Eng.

[13] Grood ES, Suntay WJ (1983). A joint coordinate system for the clinical description of three-dimensional motions: application to the knee. J Biomech Eng.

[14] Wang H, Zheng N (2010). Knee rotation and loading during spin and step turn. Int J Sports Med.

[15] Mossberg KA, Smith LK (1983). Axial rotation of the knee in women. J Orthop Sports Phys Ther.

[16] Kawakami S, Fujisawa H (2019). Kinetic analysis of tandem gait on a sine-wave-shaped walkway. J Phys Ther Sci.

[17] Koo S, Andriacchi TP (2008). The knee joint center of rotation is predominantly on the lateral side during normal walking. J Biomech.

[18] Ngai V, Wimmer MA (2009). Kinematic evaluation of cruciate-retaining total knee replacement patients during level walking: a comparison with the displacement-controlled ISO standard. J Biomech.

[19] Carman AB, Milburn PD (2006). Determining rigid body transformation parameters from ill-conditioned spatial marker co-ordinates. J Biomech.

[20] Kwon JW, Son SM, Lee NK (2015). Changes of kinematic parameters of lower extremities with gait speed: a 3D motion analysis study. J Phys Ther Sci.

